# Keeping Dairy Cows for Longer: A Critical Literature Review on Dairy Cow Longevity in High Milk-Producing Countries

**DOI:** 10.3390/ani11030808

**Published:** 2021-03-13

**Authors:** Gabriel M. Dallago, Kevin M. Wade, Roger I. Cue, J T. McClure, René Lacroix, Doris Pellerin, Elsa Vasseur

**Affiliations:** 1 Department of Animal Science, McGill University, Sainte-Anne-de-Bellevue, QC H9X 3V9, Canada; kevin.wade@mcgill.ca (K.M.W.); roger.cue@mcgill.ca (R.I.C.); elsa.vasseur@mcgill.ca (E.V.); 2 Department of Health Management, Atlantic Veterinary College, University of Prince Edward Island, Charlottetown, PE C1A 4P3, Canada; jmcclure@upei.ca; 3 Lactanet, Valacta, 555 Boul des Anciens-Combattants, Sainte-Anne-de-Bellevue, QC H9X 3R4, Canada; rlacroix@lactanet.ca; 4 Département des Sciences Animales, Université Laval, Québec, QC G1V 0A6, Canada; doris.pellerin@fsaa.ulaval.ca

**Keywords:** animal welfare, cattle husbandry, cow longevity, productive lifespan, profitability, sustainability

## Abstract

**Simple Summary:**

The ability of farms to produce milk sustainably is closely related to dairy cow longevity, i.e., the length of productive life. However, longevity is a very complex feature that depends on all the aspects of the lifespan of a cow and there is no standard definition nor metric to measure it. Measuring longevity is important because it influences the profitability and the environmental impact of farms as well as the welfare of the animals. The objectives of this paper were to review metrics used to measure longevity and describe its status among high milk-producing countries. Increasing dairy cow longevity would imply that an animal has an early age at first calving and a long and profitable productive life. Combining age at first calving, length of productive life, and margin over all (available) costs provides a complete evaluation of longevity. This paper also shows that dairy cow longevity has decreased in most high milk-producing countries over time, which confirm the concerns voiced by the dairy industry and other stakeholders. Increasing cow longevity would reduce health costs and increase cow profitability while improving both animal welfare and quality of life, contributing to a more sustainable dairy industry.

**Abstract:**

The ability of dairy farmers to keep their cows for longer could positively enhance the economic performance of the farms, reduce the environmental footprint of the milk industry, and overall help in justifying a sustainable use of animals for food production. However, there is little published on the current status of cow longevity and we hypothesized that a reason may be a lack of standardization and an over narrow focus of the longevity measure itself. The objectives of this critical literature review were: (1) to review metrics used to measure dairy cow longevity; (2) to describe the status of longevity in high milk-producing countries. Current metrics are limited to either the length of time the animal remains in the herd or if it is alive at a given time. To overcome such a limitation, dairy cow longevity should be defined as an animal having an early age at first calving and a long productive life spent in profitable milk production. Combining age at first calving, length of productive life, and margin over all costs would provide a more comprehensive evaluation of longevity by covering both early life conditions and the length of time the animal remains in the herd once it starts to contribute to the farm revenues, as well as the overall animal health and quality of life. This review confirms that dairy cow longevity has decreased in most high milk-producing countries over time and its relationship with milk yield is not straight forward. Increasing cow longevity by reducing involuntary culling would cut health costs, increase cow lifetime profitability, improve animal welfare, and could contribute towards a more sustainable dairy industry while optimizing dairy farmers’ efficiency in the overall use of resources available.

## 1. Introduction

Dairy cow longevity is the length of life of the animal, which in turn is determined by either culling decision made by the producer or death of the animal. The removal of cows from dairy herds due to old age is rare in the modern dairy industry and the economic interest associated with farm animals, which require them to achieve expected production levels, to reproduce regularly, and stay healthy [1,2], influences the farmer’s decision regarding the optimum moment to cull a cow. It is a complex decision process and a myriad of factors are to be considered by the dairy farmer [3]. Therefore, longevity is a compound feature reflecting a successful combination of many different aspects during the lifespan of a cow [4]. Dairy cow longevity is linked to the economic performance of farms, the environmental footprint of the milk industry, and the welfare status of the animals [1,5,6,7,8,9], and short cow longevity limits the achievement of a sustainable dairy industry.

The genetic potential for longevity has increased over the years [10,11,12] reflecting the inclusion of functional traits in the calculation of estimated breeding values [4]. Even though dairy cow have a life expectancy of around 20 years [13], this is rarely observed under modern commercial conditions. In Canada for example, the average age that Holstein cows die due to natural causes is 9.1 years [4]. This would represent a productive life (length of time between first calving and culling/death) of 6.8 years or about 6 lactations if an average age at first calving of 27 months is assumed [4]. This has been appointed as a problem by the dairy industry and, contrary to milk recording, there is no standardized approach to measure longevity which results in different metrics being used by different countries [14]. Increasing dairy cow longevity could be a strategy to improve the efficiency of using resources available to the dairy farmer and to produce milk with inherited sustainability.

The purpose of this critical literature review is to provide an integrated view of dairy cow longevity combined with the analysis of its status by focusing on phenotypical aspects of longevity rather than its genetic aspects. The objectives were to (1) review metrics commonly used to measure dairy cow longevity and, (2) use the most common metric to describe the status of longevity in high milk-producing countries. We hypothesized that limitations exist on current longevity metrics such as the lack of both a standard metric and reporting by dairy herd improvement (DHI) agencies or national databases. The significance of this critical review is to overcome these limitations by developing a standard methodology to estimate longevity metrics, which allow for a fair comparison between different countries, and by demonstrating that dairy cow longevity has decreased over the years in most high milk-producing countries. Addressing these two objectives will then lead us to answering the following questions: (i) should we improve dairy cow longevity? and (ii) how can we improve dairy cow longevity? and result in (iii) proposing a more comprehensive definition of cow longevity.

## 2. How Can We Measure Longevity?

The longevity of dairy cows is influenced by culling decisions made by the dairy farmer since culling ultimately defines the total length of time a cow remains in the herd. Therefore, common longevity metrics reflect culling strategies as well as the different stages of the life of a dairy cow.

### 2.1. Culling

Culling is the process of removing an animal from the herd due to death, salvage, sale, or slaughtering [2]. Apart from death, culling is a decision usually made by the dairy farmer and it is influenced by the economic interest associated with farm animals. Culling can be further classified as voluntary or involuntary based on the main reason underlying the culling decision. Voluntary culling occurs when a fertile and healthy animal is culled due to low milk production [2,15]. On the other hand, an involuntary culling happens if low milk production is not the culling reason [2,15].

**Figure 1:**
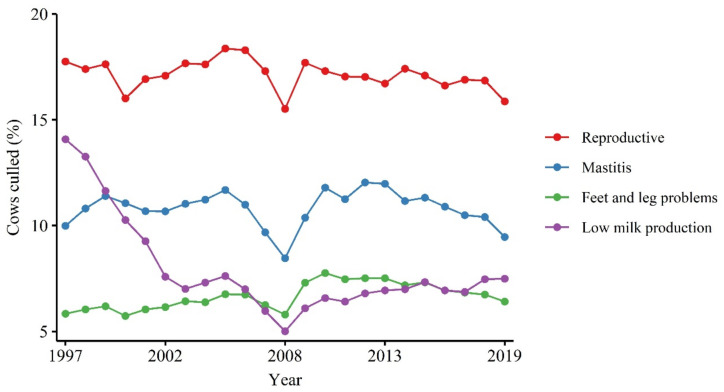
Change over time of the top four culling reasons based on the total number of cows culled with a known reported reason in Canada between 1997 and 2019 [16].

Involuntary culling accounts for most of the removal of dairy cows with known reasons. For example, in Canada, the average involuntary culling was 73.6% (Standard deviation; SD = 0.65) between 2014 and 2019, while the averages of voluntary culling and culling with unknown reason were 7.18% (SD = 0.28) and 20.7% (SD = 2.3), respectively, in the same period [16]. The high percentage of culling with unknown reason indicates the existence of limitations among producers to keep track of culling records within the farm and reporting it. Reproduction (16.8%; SD = 0.51), mastitis (10.6%; SD = 0.66), and feet and leg problems (6.88%; SD = 0.33) were the main reasons for involuntary culling during this period [16]. Similarly, infertility (20.4%), udder health (14.7%), and leg disorders (12.2%) have been reported as the main reasons for involuntary culling in Germany between 2010 and 2013 [17]. The prevalence of the main culling reasons remained stable over time. In a meta-analysis conducted by Compton et al. [18] on 51 published papers regarding 54 studies conducted in 22 different countries between 1989 and 2014, the annual incidence risk of culling due to udder and reproduction issues did not change for almost two decades starting at the mid-1980s. At the same time, there was a decrease in culling due to low milk production (voluntary culling). A similar condition was observed in Canada (Figure 1), in which the percentage of involuntary culling remained stable between 1997 and 2019 [16] for reproduction, mastitis, and feet and leg problems, while the culling for low milk production decreased up to 2008 after which it was seen a slight upwards trend. The main reason for such reduction in the voluntary culling is likely due to the genetic selection for high milk-yielding cows, which reduces the relative risk of being culled due to low milk production and is likely to continue as an objective of dairy farms [18].

The risk of culling is not constant over the life of a cow. It depends on cow factors such as lactation number, stage of lactation, milk yield, and reproductive status as well as environmental factors such as season of calving and herd-production needs [17,19,20,21,22]. Death, as well as diseases and injury, are the main reasons for culling early after the onset of a new lactation [19,20]. On the other hand, the risk of culling due to failure to reproduce and low milk production increases as the lactation progress and the highest risk is observed at later stages of the lactation [17,19,20]. Milk yield and reproduction are protective factors against culling, in which pregnant [21] and high yielding animals are less likely to be culled compared with its counterpart [19,23]. Death is mostly associated with seasonal effects in which hot seasons are associated with a greater risk of dying [19]. Cows are favored to remain in the herd if they are healthy, reproduce regularly, have functional feet, legs, and udders, and produce enough milk [1,2,13].

### 2.2. Longevity Measures

**Figure 2:**
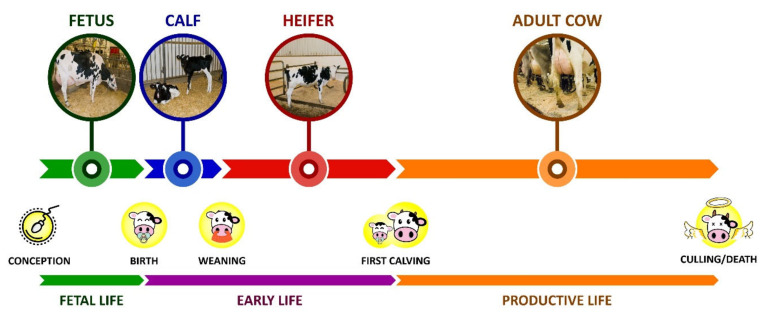
Schematic representation of the life of a dairy cow according to a chronological sequence of key events (conception, birth, weaning, first calving, and culling/death) that prompt a change to the different life status (fetus, calf, heifer, and adult cow) and respective life stages (fetal, early, and productive life). The length of the arrows is proportional to the duration of each status and stage as seen in the province of Quebec, Canada [24].

Longevity can be categorized as true, functional, and residual longevity. True longevity indicates the ability of an animal to delay culling, but it is not adjusted for milk yield [14]. Functional longevity indicates the ability of an animal to delay involuntary culling, and it is adjusted for milk yield within the herd [14]. Lastly, residual longevity represents cow longevity after adjusting it for all other traits under consideration in the breeding program [14]. Having culling or death as the endpoint and based on the different stages of the life of a dairy cow (Figure 2), different longevity metrics have been used (Table 1). These metrics can be obtained at the herd-level when they reflect the overall prevalence of animals that meet certain criteria such as the number of animals on third or greater lactation, or at the animal-level when each animal is individually evaluated.

The different longevity metrics can be classified as stayability metrics or lifetime metrics. Stayability metrics have a binary nature and indicate if a dairy cow is alive at a given moment in time [32] and can be updated as the animal grows. An example would be if the cows reach the third or greater lactation [25,26,27]. Even though such metrics do not provide a complete picture of cow longevity, one of their advantages is that they can be measured at any time [32]. On the other hand, lifetime metrics take into account the completed life stages of the animals [32]. For example, the life of a dairy cow can be split into early life (non-productive) and productive stages (Figure 2) from a production perspective. Based on that, longevity can be measured as the length of the productive life of a dairy cow [29,30]. Since lifetime metrics take into account the entire stage of life, they can only be calculated when such a stage is completed, which is one of the main limitations of such metrics.

Most lifetime metrics of dairy cow longevity do not specifically account for the early life stage (Figure 2), since they typically have first calving as the starting point. The longevity index (Table 1) is a proposed metric that overcomes such limitation by taking into account both the length of life of an animal and the length of time spent on producing milk [28]; therefore, accounting for the entire non-productive period of life (early life stage) and days dry of a dairy animal.

### 2.3. Limitation of Common Longevity Measures

There is no standard metric to measure dairy cow longevity and even though each different metric reflects an aspect of dairy cow longevity, they are not comparable since they do not have the same meaning [4]. Mark [14] estimated the correlation between longevity metrics used by different Interbull (an international network that carries on genetic analysis of livestock animals) member countries. The author reported a low correlation coefficient (0.59) and high variability (range = 0.96) among all countries evaluated, regardless of how longevity was measured/defined in each country. However, the correlation increased (0.71) and the variability decreased (range = 0.51) while analyzing only countries that used comparable longevity traits. Differences in how longevity was measured between countries could be partially the reason for low correlation and great variability in both cases, but a correlation lower than unity among countries that used comparable metrics could also be due to differences in culling reasons and trait definitions [14], which indicates a lack of standardization on measuring longevity. At the same time, the differences in the environment could be a reason for slight differences in traits among different countries as well.

## 3. What Is the Current Status of Dairy Cow Longevity and Milk Yield?

The average length of productive life, which is one of the most common longevity metrics, can be estimated based on the culling rate [13,33,34]. Since information at country level regarding culling is not available for most countries, a proxy can be estimated based on slaughtering data at country level, even though this approach would not take into account animals that died in the farm and would assume the accuracy of slaughter records reported by each country. Once this information is obtained, it can be used to evaluate the trend over time in the status of dairy cow longevity along with milk yield per animal. The following methodology was used to identify high milk-producing countries and estimate dairy cow longevity at the country level.

### 3.1. Sourcing the Information

Countries were first ranked based on total milk production to identify the ones with the highest production. As a starting point, the average total whole fresh cow milk was calculated based on 2016, 2017, and 2018 information provided by the FAO [35]. The top 21st high milk-producing countries were kept. Next, we searched for yearly official statistics publications for each of these countries regarding the total number of dairy cows, total milk production (kg), average milk yield per animal (kg), and the number of slaughtered cows. No date limit was imposed at this stage and all information available was gathered and aggregated into a single data file. For countries that reported milk production in liters it was converted to kg using the 1.03 conversion factor. For countries that did not officially report average milk yield, it was estimated by dividing the reported total milk production over the number of dairy cows (Table A1). References and official sources are presented in Table A1.

The length of productive life was estimated based on the culling rate. A proxy of the average culling rate was estimated at the country level by dividing the number of dairy cows slaughtered per year by the total number of dairy cows in each year for the countries that we were able to find both information. For countries that did not specify the number of dairy cows slaughtered, we used the number of cows slaughtered (Table A1). The inverse of the culling rate was then used as an estimation of the length of productive life [13,33,34].

**Figure 3:**
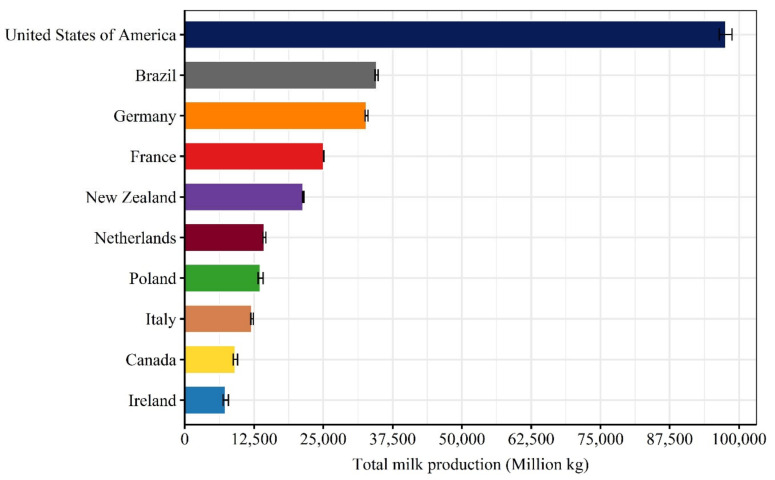
Top 10 high milk-producing countries based on total milk production averaged over the years 2016 to 2018. Columns represent the averages followed by the standard deviation (error bars). The list of countries is limited to those for which we were able to provide sufficient and reliable data on the length of productive life. Data sources are provided in Table A1.

Once the data was gathered and calculations were completed, two criteria were used in data cleaning to define its sufficiency and reliability, respectively. First, only countries that we were able to find information for at least two consecutive decades were kept for further steps. Next, information from countries in which cows had a length of productive life lower than 1.5 years (Argentina, Australia, and Mexico) in earlier decades or greater than 7 years (Turkey and United Kingdom) in recent decades were considered unreliable and excluded from further steps in this review. After cleaning, information from 10 countries remained (Figure 3; Table A1).

Linear regression was used to describe the trend over time in both milk yield and length of productive life. For milk yield, we reduced the number of observations to standardize the time window interval for all countries. Therefore, we considered only the information ranging from 1961 to 2018, which represented 96.9% of the data available after cleaning. For the length of productive life, it was not possible to establish a standard time window. For some countries, we were able to find reliable information only from more recent years while for others the collection was more extensive. The following polynomial regression model was used to describe both trends:

(1) $$Y_{j}{\;=\;\beta }_{0}{\;+\;\beta }_{1}{\mathrm{Year}}_{j}{\;+\;\varepsilon }_{j},$$

in which Y_j_ represented the milk yield per animal (kg) or length of productive life (year), β_0_ was the intercept, β_1_ was the linear regression coefficient, Year_j_ was the value observed in the jth year and ε_j_ was the residual error ~ N (0, σ^2^). Statistical significance level was set at α < 0.05.

### 3.2. Milk Yield and Longevity Over Time

**Figure 4:**
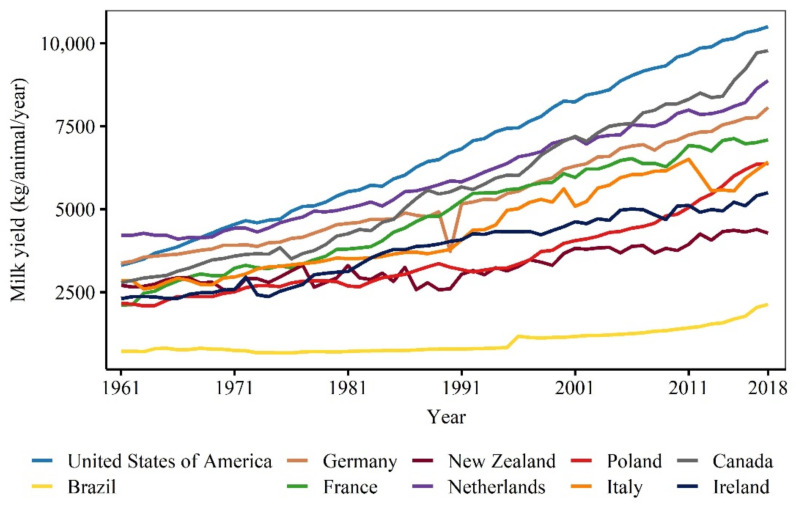
The average milk yield (kg) per animal from the top 10 high milk-producing countries over the years. The list of countries is limited to the world’s top high milk-producing countries for which we were able to provide sufficient and reliable data on the length of productive life. Data sources are provided in Table A1.

The average milk yield per animal per year increased in all the countries considered in this review (Figure 4). However, the magnitude of the increase was not the same across countries. The estimated increase ranged from 18.5 kg (Standard error; SE = 1.49) per animal per year in Brazil to 129.7 kg (SE = 1.20) kg in the United States both from 1961 to 2018 (Table 2).

Improvements in nutrition, genetics, animal health, and management of environmental factors contributed to the increase in milk yield [36,37]. However, the relative weight of such factors is likely not the same across countries. For instance, the tropical climate in Brazil limits the raising of high yielding animals such as Holstein cows, which are particularly susceptible to heat stress [38] and had their susceptibility highlighted due to intensive selection for milk production [36]. Climatic conditions are not as limiting in countries under a similar low input pasture-based production system than Brazil but located in a cooler climate zone, such as New Zealand, where climatic conditions are adverse towards production for only up to 20% of days in a year [39]. On the other hand, milk yield increase in typical indoor-housing high input systems such as in the Netherlands, United States, and Canada was achieved by intense selection of animals based on milk production instead of increasing their resistance to climatic stressors and focused on improving nutritional management and developing artificial thermal conditioning systems [36].

**Figure 5:**
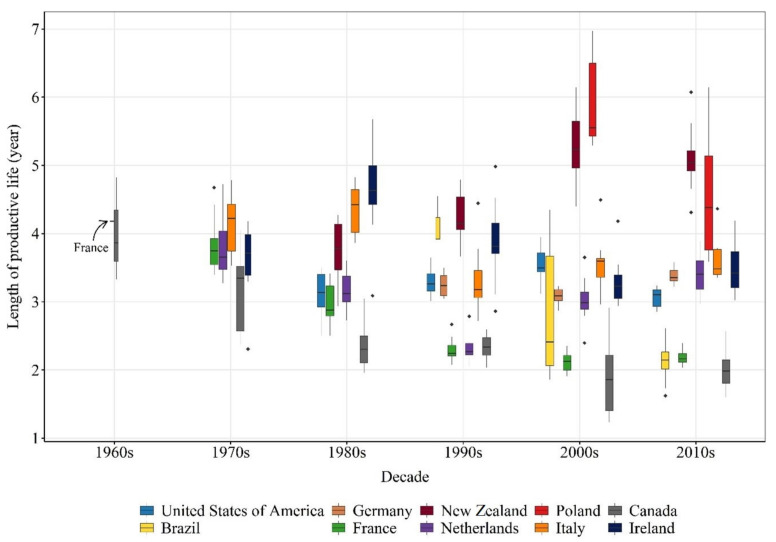
The length of productive life (year) of dairy cows from the top 10 high milk-producing countries on different decades. The relative width of each box per country within decades represents the number of observations available to generate it. The wider the box, the more observations were available. The list of countries is limited to the world’s top high milk-producing countries for which we were able to provide sufficient and reliable data on the length of productive life. Full circles (•) represent values above or bellow the interquartile range. Data sources are provided in Table A1.

Three different status of dairy cow longevity were observed in top high milk-producing countries (Figure 5). In most countries (6 out of 10), the length of productive life significantly decreased over the years, with a total estimated decrease ranging from 0.90 year in Ireland to 3.04 year in Poland (Table 3). New Zealand was the only country in which the length of productive life increased over time, with a total estimated increase of 1.85 years (Table 3). The length of productive life did not change in the United States, Germany, and the Netherlands (Table 3) with an average of 3.25 (SE = 0.09), 3.24 (SE = 0.07), and 3.14 (SE = 0.17) years, respectively.

In order to look at the relationship between milk yield and longevity, the differences in production systems need to be considered since not every country uses the same system. For instance, most herds in New Zealand are under a low input pasture-based system while in Canada and the Netherlands cows are typically housed indoors. The average milk yield per animal in New Zealand in 2018 was 2.3 and 2.1 times lower than in Canada and the Netherlands, respectively (Figure 4), which was expected since milk yield in a pasture-based system is usually lower compared to indoor-housed systems. The opposite was observed for longevity between these countries. In the 2010s decade, the average length of productive life in New Zealand was 2.5 and 1.5 times higher compared to Canada and the Netherlands, respectively (Figure 5).

The highest incidence of involuntary culling due to fertility issues and health problems such as mastitis and lameness is one of the main factors responsible for a reduction in dairy cow longevity [40]. Involuntary culling reduces the ability of dairy farmers to select animals based on production once they reach the productive life stage [41,42], forcing farmers to cull an animal that would otherwise be kept in the herd. However, such high incidence is not a reality in all farms within countries, indicating differences among farmers in their ability to keep animals healthy and comfortable for longer in the herd based on adopting management and housing practices that in turn prevent the occurrence of health problems associated with involuntary culling [40].

Differences in production systems could be associated with the longevity status of the animals in different countries. Indoor housing and high input milk production system are two of the main characteristics shared by most of the high milk-producing countries in this review in which the length of productive life decreased over time. In turn, these are also two of the main differences compared to the production system in New Zealand, where the length of productive life increased. Even though a comparison between systems regarding their effect on the main involuntary culling reasons (reproduction, mastitis, and feet and leg problems) would be inevitably confounded by milk production and animal characteristics between countries even within the same breed, it could be a starting point in exploring the reasons underlying such differences in longevity between countries.

### 3.3. Longevity and Involuntary Culling

Information on culling and culling reasons is not available at the country level for most of the high milk-producing countries covered in this review. Therefore, we rely on herd prevalence reported by epidemiological studies, which are usually conducted on a limited number of animals and farms.

#### 3.3.1. Reproduction

Failure to reproduce is the most frequent reason for involuntary culling worldwide [16,17,19] and the incidence of uterine diseases have a negative effect on animal reproduction, which could lead to a shortened longevity. Endometritis is the most prevalent uterine disease in dairy cows. Its prevalence was 27.1 or 25.1%, depending on the diagnostic method (degree of purulent vaginal discharge or cytology of the endometrium, respectively) in New Zealand [43]. In the United States, the prevalence of clinical endometritis was 15.0% [44] while the prevalence of subclinical endometritis ranged from 13.4% [44] to 53% [45]. Uterine diseases have a negative effect on animal reproduction by increasing the number of artificial inseminations per pregnancy, delaying the restart of estrous cyclicity [44], and reducing the overall pregnancy rate [45,46,47]. Therefore, cows with high longevity are likely to have a better reproductive performance, such as shorter calving interval, require a lower number of inseminations to become pregnant, and reduced number of days to first service [42]. However, having had uterine diseases do not put the cow at a greater risk of being culled if she gets pregnant [46], which demonstrate the protective effect of a positive reproduction status (being pregnant) against involuntary culling.

The reproductive performance of cows under different production systems has not been extensively studied. The reproductive health (calving difficulty, puerperal metritis, and endometritis) of seasonally bred dairy cows in a rotational grazing system tended to be better compared to cubicle housed cows in Ireland [48]. A multi-year experimental study conducted by Washburn et al. [49] at the North Carolina State University—the United States between 1995 and 1998 compared the reproductive performance of seasonally bred Jersey and Holstein cows kept under pasture or housed in a free-stall barn. Reproductive performance was measured as the percentage of pregnant animals in 75 days after the beginning of the breeding season and no difference was observed (*p* > 0.05) between systems or between breeds. However, such results need to be interpreted carefully, especially in places with climatic conditions different from those observed in these studies since animals on pasture are more susceptible to the climatic environment, which in turn can negatively affect reproduction. During summer months, the conception rate of Holstein cows kept in paddocks with little or no shade decreased by 18% in a study conducted in Florida, US [50]. In addition, oocyte quality and the development of fertilized oocytes are negatively affected by the increase in temperature observed during the summer in Holsteins cows under pasture in Louisiana, US [51]. Such negative effects are likely to be intensified in the future, given the expected changes in climate conditions.

#### 3.3.2. Mastitis

Mastitis is the most common disease in dairy cows and its occurrence varies between countries as well as within countries. The average incidence rate of clinical mastitis in Canada between November 2003 and July 2005 was 23.0%, but it ranged from 0.7 to 97.4% [52], which indicates great variability between farms. In the Netherlands, the incidence of clinical mastitis was 33.8% (95% CI = 31.7–36.1) [53]. A much lower average incidence rate of 12.7% as well as a narrowed range from 1.9 to 35.8% was observed in New Zealand between July 2004 and June 2005 [54]. In Brazil, where most of the dairy animals are on a pasture-based system similar to New Zealand, the average prevalence of clinical mastitis was 46.4%, but it ranged from 1.45 to 100% [55] while in Northern Ireland, where pasture is also largely used, the incidence was 29% between 2010 and 2015 [56].

Pasture-based systems are often associated with a lower occurrence of mastitis compared to indoor-housed cows. For instance, Jersey and Holstein cows housed in a free-stall barn had 1.8 times more cases of clinical mastitis compared to cows on pasture (*p* < 0.05), which resulted in free-stall cows having a culling rate due to mastitis eight times higher than cows on pasture in the United States [49]. Regular access to pasture was reported to be a protective factor against mastitis since it decreased the odds ratio of veterinary treated mastitis (Odds ratio; OR = 0.73, *p* < 0.05) in Austria [57]. Indoor housing was also associated with a 4.86 OR of developing subclinical mastitis during the first 41 days of lactation in Germany [58].

The cleanliness of the animals, which indicates the level of exposure to environmental pathogens, seems to be one of the reasons for such a protective factor of pasture. The cleanliness of stalls in a free-stall barn was positively correlated with the hygiene scores for udder [59], which in turn was associated with increased somatic cell count [60,61]. Cows that had access to pasture were 3.75 (SE = 1.89; *p* < 0.05) times less likely to be dirty compared to cows that did not in Danish dairy farms [62]. However, the cleanliness of cows on pasture or at outdoor paddocks is directly influenced by climatic conditions, which in the rainy season is associated with dirtier cows while the opposite is observed during the dry season [63]. In addition, the occurrence of mastitis is associated with hygiene practices and improving those are a low-cost solution that improves animal performance [64] and the incidence of mastitis. In indoor housing, increasing the frequency of cleaning the barns could be a strategy to reduce the level of exposure to pathogens, since that cleaning the floors more than 4 times per day was associated with a reduction in clinical mastitis incidence (OR = 0.77; 95% CI = 0.62–0.96; *p* < 0.05) in the Netherlands [65].

#### 3.3.3. Feet and Leg

The occurrence of lameness is lower in cows on pasture compared to indoor-housed cows. The prevalence in New Zealand is 8.1% [66] compared to 22.2% and 24.6% in Canada [67] and the United States [68], respectively. Such difference between systems is even present within the same countries. In the UK, Haskell et al. [69] reported that zero-grazing farms had 2.6 more lame cows compared to grazing farms while in the United States, Adams et al. [70] reported that in farms where cows were primarily housed in free-stall barns had an 6.9 (SE = 0.60) greater incidence density ratio of severely lame cows than in farms where cows were kept mainly in pasture. Concrete floor is a risk factor in increasing the incidence of claw lesions [71] and lameness [71,72]. To that end, access to pasture could be beneficial because it has been associated with improving hoof health, healing of lesions, and decreasing the incidence of lameness [71,73,74]. The low incidence of lameness in New Zealand could result in a reduction of involuntary culling due to feet and leg problems and potentially increase the longevity of dairy cows in this country compared to Canada and United States where pasturing cows is seldom practiced. However, in addition to information on culling not being available at the country level for most countries, failure in detecting lameness by farmers is a limiting factor in using herd prevalence as a proxy for culling reason. The prevalence of lameness is 3 to 4 times higher than that estimated by farmers [66,68,75]. In addition, lame cows are not necessarily culled since they can be treated if the producer chooses to do so. Lameness is also associated with negative reproductive performance and milk production [76], which in turn might be the reason reported for culling by the farmer.

By itself, the pasture-based system is not responsible for reducing the prevalence of lameness in dairy cows. An overall prevalence of 39% was reported by Thompson et al. [77] while evaluating 252 dairy cows from six pasture-based herds in the southern region of Brazil, which is higher than that reported in indoor housed animals in Canada (22%) [67] and US (24.6%) [68]. Environmental conditions and management practices such as the amount of rainfall, condition of tracks to pasture, poor hygiene, and human–animal relationship are important factors associated with lameness in pasture-based farms [78,79]. In addition, most of the time, farmers can only report one reason for culling, and feet and leg issues may not be the primary reason for culling due to their failure to detect lame animals [66,68,75].

## 4. Should We Improve Dairy Cow Longevity?

Short longevity poses a threat to the sustainability of the dairy industry since it is associated with financial losses on farms, increased environmental footprint of milk production, and welfare issues for the animals, which in turn is a growing social concern among consumers [1,5,6,7,8,9]. Therefore, improving dairy cow longevity would contribute to achieving a more sustainable industry, since it would have a positive effect towards the three pillars of sustainable agriculture: economic profit, environmental impact, and social concerns.

### 4.1. Economic Profit

For a dairy farm to be profitable, dairy cows need to be able to reproduce regularly, maintain high milk production, and do not fall ill for many years [80]. Therefore, increasing the length of productive life is a potential option to improve the profitability of the dairy activity [7]. In fact, it is the second most economically important trait in dairy cows, while milk yield is the first most important trait [81]. Short longevity indicates that animals are not expressing their maximum potential for productivity and profitability, since dairy cows become profitable at their third lactation due to high costs associated with the early life non-productive stage [8,9]. In addition, more first and second lactation cows are culled as culling rate increases [82], which decreases animal longevity and reduces the profitability of the system. Overall, the most common reason for culling of first and second lactation cows is reproduction issues while death is the most common reason for third and greater lactation cows [19]. During the initial third of the lactation, first calving cows are more likely to be culled due to low milk production and milkability while second lactation animals are culled due to the incidence of metabolic and other diseases [17]. However, higher risk of culling due to failure to reproduce is observed in the final third of the lactation for both first and second lactation cows [17]. With increasing longevity by decreasing the culling of animals in the beginning of their productive life, there will be a high number of cows on more profitable lactations in the herd and the replacement cost per day will be relatively reduced since it would be split into more lactations [1].

Having a greater proportion of mature cows because of increased longevity would reduce the number of replacement heifers required to achieve the same milk production since mature cows have a relatively higher milk yield compared to young animals. This is particularly relevant under a supply management system such as the one present in Canada [4], where profitability is associated with increased efficiency of using the resources available and reducing input costs rather than increasing milk production. However, it would allow for the commercialization of extra heifers [5,10,42] and potentially increasing this additional source of income. In addition, increasing longevity by reducing involuntary culling would improve lifetime profit [1] especially given the negative economic impact of factors underlying health problems associated with involuntary culling [64].

Even though longer longevity alone does not assure an increase in profitability, a farm with short longevity due to a high involuntary culling and its associated diseases is not likely to be profitable either [34]. The adoption of management practices and technologies to improve cow health and longevity is essential to achieve a profitable dairy industry in the future, which is a key factor in achieving sustainability [83,84].

### 4.2. Environmental Impact

Increasing longevity would reduce the environmental toll of the dairy industry. Longer longevity would reduce the required number the replacement heifers needed on a farm, which contribute with 21 to 26% of the total enteric emission of methane in a herd [85]. At the same time, it would reduce the proportional emission from replacement heifers. Assuming an age at first calving of 28 months, Knapp et al. [86] estimated that increasing the length of productive life from 2.5 years (40% culling rate) to 4.0 years (25% culling rate) would reduce by 9.5% the enteric emission contribution of replacement heifers. In addition, methane emission per animal does not increase as the animal gets older [87]. In fact, an increase in the length of productive life was associated with a decrease in methane emission per kg of milk corrected for fat and protein [7], which contributes to decreasing the footprint associated with milk production [6] and supports the argument that increasing dairy cow longevity would decrease the environmental burden of the dairy industry.

### 4.3. Social Concerns

Early age at culling is a growing concern among consumers [88], especially because cow longevity is a global indicator of animal welfare since higher cow longevity indicates that the animal biological functions and health are not impairing the length of its life [89]. In addition, the health issues associated with the most common reasons for involuntary culling reported by dairy farmers bring into question the welfare conditions and ethical concerns towards dairy farming [34].

The high incidence of involuntary culling due to reproduction problems [16,17,19] might hide underlying health problems. For instance, the occurrence of reproductive diseases [44,45,46,47] as well as lameness [76] and mastitis [90] have a negative effect on the ability of an animal to get pregnant and might result in animals being culled with failure to reproduce as the reported reason. However, increased longevity is not always associated with improved cow welfare. The incidence of health problems is directly associated with a poor cow welfare status and older animals are more likely to develop health problems such as lameness [91] and mastitis [57] as well as body injuries [92]. Therefore, the increase in cow longevity should be the result of improving the ability of dairy farmers to keep animals healthy and comfortable, which in turn improves the overall animal welfare status.

The main reported reasons for involuntary culling imply a lower status of animal welfare, which was the primary issue raised by consumers towards an ideal dairy farm [93]. Leg problems such as lameness or foot disorders are considered the most detrimental condition on animal welfare [89,94], while peripartum problems such as dystocia and retained placenta, which are associated with decreased reproductive performance, can be life-threatening or occur because of chronic stressful conditions [95].

Animal welfare becomes economically important to consumers once they attach importance to animal suffering [96]. Consumers from Europe [97] and the United States [98] indicated a willingness to pay more for animal-based products obtained from farms with high welfare status. European consumers also stated that products imported from other countries should be subject to the same level of welfare standards that are imposed on farmers in the European Union [97], which indicates that animal welfare could become a commercial barrier between countries [96,99]. Even though willingness to pay does not always translate into action, it would be prudent to expect that future demand for higher welfare status of dairy cattle among consumers will remain, including a demand for longer longevity [34].

## 5. How Can We Improve Dairy Cow Longevity?

Dairy cow longevity is the outcome of decisions made by dairy farmers throughout the life of the animal, which dictates the moment and the reason a cow is culled. It is a dynamic process where multiple factors and their interactions are to be considered by the farmer [3]. Therefore, all aspects of a cow’s life need to be considered to reduce the rate of involuntary culling and increase longevity [1]. In addition, most lifetime metrics of longevity only become available once the animal is culled. To overcome such limitation, a currently rich area for research is the identification of metrics available earlier in the life of the animal that, in turn, are correlated with lifetime longevity metrics available later in life.

### 5.1. Early Life Indicators

#### 5.1.1. Age at First Calving and Its Association with Longevity Metrics

Age at first calving (AFC) is a relatively early life metric, which has been extensively studied. The average AFC between high milk-producing countries is presented in Table 4, which ranged from 24.6 in the Netherland to 32.6 in Brazil. Age at first calving is associated with the ability of cows to remain in the herd and avoid culling, since animals that calved for the first time at a young age are less likely to be culled early during the productive life. Based on information from 437 herds across the United Kingdom, Sherwin et al. [100] reported that cows with an AFC greater than 30 months were 1.71 times more likely (*p* < 0.05) of being culled compared to animals with an AFC of 23–24 months. In another study conducted on 7768 Holstein heifers born between 2004 and 2006 in Spain, Bach [101] reported that heifers which finished their first lactation had an average AFC of 23.8 months compared with an average AFC of 24.2 months of animals that did not.

Age at first calving is also associated with the length of productive life. Swedish dairy cows that had an AFC of 27–28 months were 1.1 times more likely (*p* < 0.05) to have a shorter length of productive life compared to animals with an AFC younger than 25 months [21]. Similar results were reported in a study carried out by Nilforooshan and Edriss [102] using production and pedigree data from Iranian Holstein cows collected between 1991 and 2011 from 45 herds, in which the length of productive life decreased as AFC increased (*p* < 0.05). The opposite was reported in a study using records from a single Australian farm from 1992 to 2005, in which animals with an AFC greater than 36 months had a longer length of life (*p* < 0.05) compared to animals calving for the first time between less than 24 to 36 months [28]. However, the opposite was observed for the longevity index in the same study (*p* < 0.05), indicating that animals with an older AFC had a longer length of life, possibly because the animals were inseminated older for the first time since the number of parities per lifetime did not differ (*p* = 0.28) between animals [28].

**Table 4:** Age at first calving (month) and length of life (year) of dairy cows in high milk-producing countries. The list of countries is limited to the world’s top high milk-producing countries for which we were able to provide sufficient and reliable data on the length of productive life and recent age at first calving from official milk recording agencies.

Country	Year	Recorded Herds	Recorded Cows	Percentage of Recorded Cows ^1^	Breed	Recorded 1st Lactations	Age at First Calving	Reference	Length of Life ^2^
United State of America	2019	2140	---	---	7 different dairy breeds	---	25.5 ^4^	AgSource [103]	4.98
Brazil	2018	334	15,459 ^3^	0.09	Girolando (Holstein/Gir crossbreed)	12,384	32.6	GIROLANDO [104]	4.34
Germany	2018	---	---	---	15 different dairy breeds	967,996	27.7	BRS [105]	5.67
France	2018	35,253	2,437,250	69.0	20 different dairy breeds	776,679	30.0 ^5^	idele [106]	4.59
Italy	2019	15,316	1,351,442	72.7	30 different dairy breeds	321,298	27.3 ^5^	AIA [107]	5.69
Poland	2019	20,644	820,653	37.1	12 different dairy breeds	250,159	26.7	PFHBIPM [108]	6.23
Netherlands	2019	14,367	1,459,287	91.9	Black-and-white dairy breeds, Red-and-white dairy breeds, and others	---	24.6	CRV [12]	5.88
Ireland	April 2020	---	1,599,498	---	---	---	26.5	ICBF—Irish Cattle Breeding Federation [109]	6.39 ^7^
Canada	2019	7063	658,311	68.0	---	---	25.0 ^6^	CDIC [110],Lactanet [111,112]	3.89

^1^ Relative to the total number of cows in the country; ^2^ Age at first calving plus the length of productive life from each country (Figure 5); ^3^ Number of lactations recorded; ^4^ Average of averages weighted over the number of herds by breed, since the number of recorded 1st lactations was not available; ^5^ Average of averages weighted over the number of recorded 1st lactations; ^6^ Median; ^7^ Estimated using the length of productive life of 2018.

#### 5.1.2. Other Early Life Indicators and Their Association with Longevity Metrics

Looking at longevity with AFC as the starting point overlooks early life (Figure 2) management practices and decisions made by the dairy farmer and their effect on the productive life of dairy cows. Even though it has received much less attention in the literature, there is an increasing interest in the subject. Housing calves from 3 to 7 months in litter pens with ≤12 calves resulted in a median increase (*p* < 0.05) of 18.2 months in the survival time compared to calves housed in slatted pens with >7 calves [21]. The age in which the animal first consumed 0.91 kg/d of grain (dry matter basis) was positively associated with the age when removed from the herd [113]. Additionally, housing automatically fed calves in small groups (6–9 calves) was associated with a higher growth rate (0.022 cm/day, about 40 g/day, *p* < 0.05) compared to calves housed in larger groups (12-18 calves; Svensson and Liberg [114]). In turn, the higher the average daily gain (ADG) of weight in different ages before the first calving, the younger (*p* < 0.05) the AFC [115]. In pasture-based dairy herds, Chuck et al. [116] reported a positive association (*p* < 0.05) between ADG from 1 month of age to first breeding on cumulative milk, fat, and protein yield at 100 and 250 days in milk in primiparous. Average daily gain from birth to weaning was also negatively associated (*p* < 0.05) with the occurrence of veterinary treated cases of mastitis from 7 to 30 days post-partum in primiparous cows [117].

Health events in early life, the season of birth, and inbreeding are associated with cow longevity. The occurrence of severe calfhood respiratory disease was associated with a 12% increase (*p* < 0.05) in the calving interval of Swedish Red dairy cows [118]. Fall- and winter-born calves had a higher 8-week calf starter intake (48.3 kg vs. 42.75 kg), ADG (0.66 vs. 0.625 kg/d), and body weight (77.5 vs. 75.0 kg) compared to spring- and summer-born calves (*p* < 0.05) [119]. While using Dairy Herd Improvement (DHI) data between 1980 and 2004 from Canadian Holstein cows (n = 1977311), Sewalem et al. [120] reported that animals with an inbreeding coefficient of 6.25 to 12.5% were 1.14 times more likely to have a shorter length of productive life, with the likelihood increasing as high as 1.51 times with inbreeding coefficient ≥ 25.0%.

#### 5.1.3. Fetal Life and Its Association with Longevity Metrics

Birth conditions are other less explored factors. The effect of complications during calving on dam longevity is well described in the literature. For instance, Holstein cows that require a hard pull and surgery during calving were 1.27 and 1.92 times more likely of being culled (*p* < 0.05), respectively compared to animals with unassisted calving [31]. However, the effect on offspring longevity has been less studied. A study conducted by Heinrichs and Heinrichs [113] on 21 dairy farms located in Pennsylvania, US reported that delivery scores indicating unassisted, easy pull, hard pull, mechanical extraction, or cesarean section were not associated (*p* = 0.11) with age when the offspring were removed from the herd. However, more studies are needed.

Fetal programming (the effect of dam conditions during conception and gestation on offspring performance) has been more extensively studied in beef [121,122] compared with dairy animals, but a few studies demonstrated associations between the dam’s conditions on outcomes observed later in the life of the offspring. For instance, dam’s intrauterine conditions associated with milk production seem to have an effect on offspring performance and survival [123], even though metabolic stress due to milk production might have a stronger effect than dam milk production alone [124]. In addition, high milk urea nitrogen is associated with decreased fertility in dairy cows [125,126] and have a negative effect on early stages of oocyte development [127,128]. The longevity of calves originated from oocytes of cows with high milk urea nitrogen before ovulation has not been studied and accounting for the dam condition during pregnancy might be a possibility to improve offspring longevity [129].

### 5.2. Lack of Space and Quota Constraints

Dairy cow longevity is not only influenced by intrinsic cow factors, but also by extrinsic factors such as availability of space in the farm as well as market characteristics, which could also influence the involuntary culling. In a situation where there is a surplus of heifers, farmers would decide to cull animals to make space for heifers that just calved [13]. In places under a supply management system such as in Canada [4], it would be difficult to accommodate the increase in milk production as a result of having more cows in more productive lactations [5] as a result of increased longevity or when producers have contracts with dairy processors that limit the amount of milk they can deliver. Both conditions would influence decreasing the rate of involuntary culling and increasing longevity.

A possible alternative would be to combine the use of sexed semen with extending the lactation of high-yielding cows by increasing the voluntary waiting period. Such a strategy would reduce the frequency that cows undergo the beginning of the lactation, which is associated with greater risk for involuntary culling due to death and diseases [17,19,20]. At the same time, the use of sexed semen would reduce the negative effect of extended lactation on the genetic return [130]. Extending the duration of lactation of high yielding animals was also shown to have no negative effect on the gain of body condition score, udder health, milk production, and culling [131] while improving their reproductive performance [132].

## 6. Proposing a More Comprehensive Definition of Cow Longevity

Contrarily to milk and milk components, dairy cow longevity is neither routinely measured nor reported. This could be partly justified by the lack of a sound definition of the term and, as a result, the nonexistence of a standard metric designed to cover all aspects outlined in the definition. The definition of longevity should take into account the health, reproductive performance, and milk production of any given animal during its entire lifespan, which in turn are key factors associated with culling [16,17,18] and the profitability of the dairy industry [1,2,13,80]. As much as possible, the definition should allow for the use of metrics that are already routinely collected from either farms or DHI agencies, making it easy to be implemented and to increase the chances of being widely adopted. To that end, dairy cow longevity could be defined as an animal having an early age at first calving and a long productive life spent under profitable levels of milk production.

**Figure 6:**
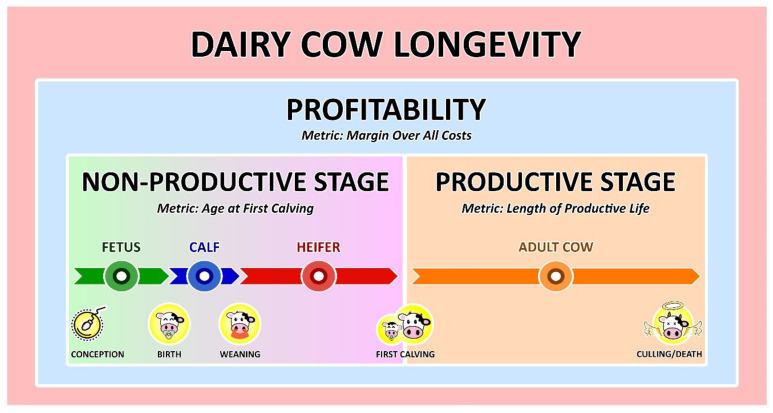
Relationship between the concepts of profitability, non-productive and productive life stages of dairy cows for a more comprehensive definition of cow longevity along with proposed metrics representing each respective concept.

This definition covers both early life conditions and the stayability of the animal once it reaches the lactating herd as well as its overall health and quality of life. Assuming that an animal would be inseminated for the first time as soon as it is ready, early age at first calving would indicate that the animal was raised under healthy and favorable early life conditions. Next, a long and profitable productive life would imply that the animal produced enough milk to justify keeping it under milking, reproduced regularly avoiding a potential extension of the lactation to unprofitable levels or unnecessarily long dry periods, and maintained good health since the incidence of health issues are directly linked with reproduction failures and reduction in milk production. Age at first calving, length of productive life, and margin over all costs are metrics that could be used as indicators of early life conditions, length of life, and profitability, respectively. Combined, they would provide a more comprehensive approach to measure dairy cow longevity (Figure 6).

## 7. Conclusions

The current metrics available to measure longevity often starts at the first lactation, overlooking early life management practices and decisions made by the dairy farmer before that point. To overcome such limitation, first, we propose that dairy cow longevity should be defined as an animal having an early age at first calving and a long productive life spent under profitable levels of milk production. Next, a combination of the metrics age at first calving, length of productive life, and margin over all (available) costs would provide a more comprehensive evaluation of longevity and cover all aspects of the definition.

By using a standard methodology, this critical literature review confirms the concerns raised by the dairy industry and other stakeholders that dairy cow longevity has decreased in most high milk-producing countries. Early life indicators are needed to support farmers in the early selection of animals that are more likely to reach their maximum potential. Increasing cow longevity due to a reduction in involuntary culling would reduce health costs, increase cow lifetime profitability, improve animal welfare and quality of life, and contribute towards a more sustainable dairy industry by producing milk with inherited sustainability while optimizing dairy farmers’ efficiency in the use of resources.

## Figures and Tables

**Table 1:** Dairy cow longevity metrics commonly used.

Measure	Unit	Time Frame	Description	Reference
Lactation	Count	First calving to culling/death	Cumulative number of lactations	Essl [1]
3+ lactation	Herd prevalence	The number at a given point	Percentage of cows on the third or greater lactation	Villettaz Robichaud et al. [25],Villettaz Robichaud et al. [26],Villettaz Robichaud et al. [27]
Culling rate	Herd prevalence	The number at a given point	Percentage of culling	Villettaz Robichaud, Rushen, de Passillé, Vasseur, Haley, Orsel and Pellerin [25],Villettaz Robichaud, Rushen, de Passillé, Vasseur, Orsel and Pellerin [26],Villettaz Robichaud, Rushen, de Passillé, Vasseur, Haley and Pellerin [27]
Length of life	Year	Birth to culling/death	Length of time between birth and culling	Haworth et al. [28]
Length of productive life	Year	First calving to culling/death	Length of time between first calving and culling	Ducrocq [29],Schneider et al. [30]
Functional longevity	Rank	First calving to culling/death	Length of productive life adjusted for within-herd milk production level	Sewalem et al. [31]
Longevity index	%	Birth to culling/death	Lifetime days in milk divided by length of life	Brickell and Wathes [5],Haworth, Tranter, Chuck, Cheng and Wathes [28]

**Table 2:** The linear trend of milk yield (kg) per animal per year for each country between 1961 and 2018. The list of countries is limited to the world’s top high milk-producing countries for which we were able to provide sufficient and reliable data on the length of productive life.

Country	Model ^1^		R ^2,3^	RSE ^4^	*p*-Value ^5^
	Intercept ^2^	Year ^2^			
United States of America	2941.6 *** (40.6)	129.7 *** (1.20)	0.99	152.5	<0.001
Brazil	451.2 *** (50.4)	18.5 *** (1.49)	0.73	189.5	<0.001
Germany	2904.6 *** (83.4)	81.4 *** (2.46)	0.95	313.4	<0.001
France	2103.8 *** (56.8)	91.9 *** (1.68)	0.98	213.6	<0.001
New Zealand	2419.1 *** (70.9)	29.0 *** (2.09)	0.77	266.7	<0.001
Poland	1603.6 *** (107.4)	65.3 *** (3.17)	0.88	403.8	<0.001
Italy	2200.5 *** (91.3)	72.5 *** (2.69)	0.93	343.1	<0.001
Canada	2081.3 *** (84.1)	120.7 *** (2.48)	0.98	316.3	<0.001
Ireland	2035.0 *** (53.3)	59.9 *** (1.57)	0.96	200.5	<0.001

^1^ *** = *p*-value < 0.01; ^2^ Estimate (Standard error); ^3^ R2 = Coefficient of determination; ^4^ RSE = Residual standard error; ^5^ Model significance.

**Table 3:** The linear trend of the length of productive life (year) in each country. The list of countries is limited to the world’s top high milk-producing countries for which we were able to provide sufficient and reliable data on the length of productive life.

Country	Year	Model ^1^		R ^2,3^	RSE ^4^	*p*-Value ^5^
		Intercept ^2^	Year ^2^			
United States of America	1980–2019	3.25 *** (0.10)	0.0004 ^NS^ (0.004)	0.0003	0.30	0.92
Brazil	1997–2018	4.06 *** (0.24)	−0.12 *** (0.02)	0.67	0.55	<0.001
Germany	1993–2019	3.11 *** (0.07)	0.01 ^NS^ (0.004)	0.13	0.18	0.06
France	1968–2019	3.89 *** (0.10)	−0.04 *** (0.003)	0.76	0.37	<0.001
New Zealand	1982–2019	3.69 *** (0.19)	0.05 *** (0.01)	0.48	0.56	<0.001
Poland	2003–2019	6.81 *** (0.25)	−0.19 *** (0.02)	0.79	0.50	<0.001
Italy	1970–2019	4.26 *** (0.14)	−0.02 *** (0.005)	0.22	0.49	<0.001
Canada	1967–2019	3.38 *** (0.13)	−0.03 *** (0.004)	0.57	0.47	<0.001
Ireland	1974–2019	4.28 *** (0.21)	−0.02 * (0.01)	0.14	0.70	0.01

^1 NS^ = Not significant, * = *p*-value < 0.10, *** = *p*-value < 0.01; ^2^ Estimate (Standard error); ^3^ R2 = Coefficient of determination; ^4^ RSE = Residual standard error; ^5^ Model significance.

## Data Availability

The data used in this study are available from the corresponding author, G.M.D.

## Appendix A

**Table A1:** The sources of official statistics published by the top 21 high milk-producing countries regarding the number of dairy cows, total milk production, average milk yield per animal per year, and the number of slaughtered cows as well as data availability information.

Country	Number of Dairy Cows	Total Milk Production	Milk Yield	Number of Slaughtered Cows	Specify the Dairy Category
United States of America	FAO [35], USDA [133]	USDA [133]	USDA [133] and calculated	USDA [134]	Yes
India	---	---	---	Illegal to slaughter cows in most parts of the country [135]	---
Brazil	FAO [35], IBGE [136]	FAO [35], IBGE [137]	Calculated	IBGE [138]	No
Germany	Destatis [139]	BZL [140], eurostat [141]	BZL [140] and calculated	Destatis [142]	No
China, mainland	---	---	---	Reports the number of slaughtered cattle and buffaloes combined and does not specify cows	---
Russian Federation	---	---	---	Reports only the total live weight of slaughtered cattle and does not specify cows	---
France	eurostat [143]	eurostat [141]	Calculated	eurostat [144]	No
New Zealand	FAO [35], NZ.Stat [145], LIC & DairyNZ [146]	FAO [35], LIC & DairyNZ [146]	LIC & DairyNZ [146] and calculated	NZ.Stat [147]	No
Turkey	---	---	---	Only available for years 2015 to 2019 [144]	---
Pakistan	---	---	---	Information not freely available	---
United Kingdom	---	---	---	Not reliable over the years [144]	---
Poland	FAO [35], eurostat [143]	FAO [35], eurostat [141]	Calculated	eurostat [144]	No
Netherlands	eurostat [143]	eurostat [141]	Calculated	eurostat [144]	No
Mexico	FAO [35]	FAO [35], SIACON [148]	Calculated	USDA [149]	No
Italy	eurostat [143]	eurostat [141]	Calculated	eurostat [144]	No
Argentina	FAO [35]	MAGyP [150]	Calculated	MAGyP [151]	No
Ukraine	---	---	Calculated	Reports the number of slaughtered cattle	---
Uzbekistan	---	---	Calculated	Reports not available	---
Australia	---	---	---	Reports the number of slaughtered cows and heifers together	---
Canada	Statistics Canada [152]	Statistics Canada [153]	Calculated	USDA [149]	No
Ireland	FAO [35], eurostat [143]	eurostat [141]	Calculated	eurostat [144]	No
