# Erratum: Dallago et al. Keeping Dairy Cows for Longer: A Critical Literature Review on Dairy Cow Longevity in High Milk-Producing Countries. *Animals* 2021, *11*, 808

**DOI:** 10.3390/ani11102958

**Published:** 2021-10-14

**Authors:** Gabriel M. Dallago, Kevin M. Wade, Roger I. Cue, J T. McClure, René Lacroix, Doris Pellerin, Elsa Vasseur

**Affiliations:** 1 Department of Animal Science, McGill University, Sainte-Anne-de-Bellevue, QC H9X 3V9, Canada; kevin.wade@mcgill.ca (K.M.W.); roger.cue@mcgill.ca (R.I.C.); elsa.vasseur@mcgill.ca (E.V.); 2 Department of Health Management, Atlantic Veterinary College, University of Prince Edward Island, Charlottetown, PE C1A 4P3, Canada; jmcclure@upei.ca; 3 Lactanet, Valacta, 555 Boul des Anciens-Combattants, Sainte-Anne-de-Bellevue, QC H9X 3R4, Canada; rlacroix@lactanet.ca; 4 Département des Sciences Animales, Université Laval, Québec, QC G1V 0A6, Canada; doris.pellerin@fsaa.ulaval.ca

In the original article [1] there was a mistake in the published version of Tables 2 and 3; information about The Netherlands was missing in both Tables 2 and 3. The corrected Table 2 and Table 3 appear below. The authors apologize for any inconvenience caused and state that the scientific conclusions are unaffected. The original article has been updated.

The authors would like to apologize for any inconvenience caused. The change does not affect the scientific results. The manuscript will be updated, and the original will remain online on the article webpage.

## Figures and Tables

**Table 2:** The linear trend of milk yield (kg) per animal per year for each country between 1961 and 2018. The list of countries is limited to the world’s top high milk-producing countries for which we were able to provide sufficient and reliable data on the length of productive life.

Country	Model ^1^		R^2 3^	RSE ^4^	*p*-Value ^5^
	Intercept ^2^	Year ^2^			
United States of America	2941.6 *** (40.6)	129.7 *** (1.20)	0.99	152.5	<0.001
Brazil	451.2 *** (50.4)	18.5 *** (1.49)	0.73	189.5	<0.001
Germany	2904.6 *** (83.4)	81.4 *** (2.46)	0.95	313.4	<0.001
France	2103.8 *** (56.8)	91.9 *** (1.68)	0.98	213.6	<0.001
New Zealand	2419.1 *** (70.9)	29.0 *** (2.09)	0.77	266.7	<0.001
The Netherlands	3485.8 *** (61.2)	84.3 *** (1.80)	0.97	230.0	<0.001
Poland	1603.6 *** (107.4)	65.3 *** (3.17)	0.88	403.8	<0.001
Italy	2200.5 *** (91.3)	72.5 *** (2.69)	0.93	343.1	<0.001
Canada	2081.3 *** (84.1)	120.7 *** (2.48)	0.98	316.3	<0.001
Ireland	2035.0 *** (53.3)	59.9 *** (1.57)	0.96	200.5	<0.001

^1^ *** = *p*-value < 0.01; ^2^ Estimate (standard error); ^3^ R^2^ = Coefficient of determination; ^4^ RSE = Residual standard error; ^5^ Model significance.

**Table 3:** The linear trend of the length of productive life (year) in each country. The list of countries is limited to the world’s top high milk-producing countries for which we were able to provide sufficient and reliable data on the length of productive life.

Country	Year	Model ^1^		R^2 3^	RSE ^4^	*p*-Value ^5^
		Intercept ^2^	Year ^2^			
United States of America	1980–2019	3.25 *** (0.10)	0.0004 ^NS^ (0.004)	0.0003	0.30	0.92
Brazil	1997–2018	4.06 *** (0.24)	−0.12 *** (0.02)	0.67	0.55	<0.001
Germany	1993–2019	3.11 *** (0.07)	0.01 ^NS^ (0.004)	0.13	0.18	0.06
France	1968–2019	3.89 *** (0.10)	−0.04 *** (0.003)	0.76	0.37	<0.001
New Zealand	1982–2019	3.69 *** (0.19)	0.05 *** (0.01)	0.48	0.56	<0.001
The Netherlands	1970–2019	3.38 *** (0.17)	−0.01 ^NS^ (0.01)	0.05	0.59	0.12
Poland	2003–2019	6.81 *** (0.25)	−0.19 *** (0.02)	0.79	0.50	<0.001
Italy	1970–2019	4.26 *** (0.14)	−0.02 *** (0.005)	0.22	0.49	<0.001
Canada	1967–2019	3.38 *** (0.13)	−0.03 *** (0.004)	0.57	0.47	<0.001
Ireland	1974–2019	4.28 *** (0.21)	−0.02 * (0.01)	0.14	0.70	0.01

^1^ NS = Not significant, * = *p*-value < 0.10, *** = *p*-value < 0.01; ^2^ Estimate (standard error); ^3^ R^2^ = Coefficient of determination; ^4^ RSE = Residual standard error; ^5^ Model significance.
